# Survival of elderly people living with the human immunodeficiency virus in a municipality in Northeast Brazil: a retrospective cohort, 2006-2021

**DOI:** 10.1590/S2237-96222025v34e20240071.en

**Published:** 2025-03-31

**Authors:** Julianne Damiana da Silva Vicente, Cristine Vieira do Bonfim, Jessyka Mary Vasconcelos Barbosa, Vanessa de Lima Silva, Albanita Gomes da Costa de Ceballos, Gabriella Morais Duarte Miranda

**Affiliations:** 1 Universidade Federal de Pernambuco, Programa de Pós-Graduação em Saúde Coletiva, Recife, PE, Brazil; 2 Fundação Joaquim Nabuco, Diretoria de Pesquisas Sociais, Recife, PE, Brazil; 3 Ministério da Saúde, Brasília, DF, Brazil; 4 Universidade Federal de Pernambuco, Programa de Pós-graduação em Gerontologia, Recife, PE, Brazil; 5 Universidade Federal de Pernambuco, Programa de Pós-graduação em Saúde Coletiva, Recife, PE, Brazil

**Keywords:** Survival Analysis, HIV infections, Elderly, Health Profile, Cohort Studies, Análisis de Supervivencia, Infecciones por VIH, Anciano, Perfil de Salud, Estudios de Cohortes

## Abstract

**Objective:**

To characterize the survival of elderly people with HIV infection who had follow-up at a reference service in Jaboatão dos Guararapes, Pernambuco, Brazil.

**Methods:**

This is a retrospective cohort of elderly people who began follow-up between 2006 and 2021. Kaplan-Meier survival analysis was used. The Cox model was applied to calculate the hazard ratio (HR) and 95% confidence interval (95%CI) of survival based on the study variables.

**Results:**

116 elderly people were analyzed, the majority (n=83) were 60-69 years old and were diagnosed after the age of 60 (n=45). Risk of death was higher among elderly people who were hospitalized during the study period (HR 4.83; 95%CI 1.07; 21.79). Nine people died from HIV-related causes and average survival time was 76.5±48.5 months. In the first year of the study, probability of survival was greater than 96%.

**Conclusion:**

Survival varied from less than one month to more than 195 months. Among the sociodemographic and clinical factors studied, only hospitalization showed significant association with occurrence of deaths among the elderly. Although the study was carried out in just one service, these results can contribute to guiding care strategies for elderly people living with HIV.

## Resumen

**Objetivo:**

Caracterizar la supervivencia de personas mayores con infección por VIH, con seguimiento en un servicio de referencia en Jaboatão dos Guararapes, Pernambuco, Brasil.

**Métodos:**

Se trata de una cohorte retrospectiva de personas mayores que iniciaron seguimiento entre 2006 y 2021. Se utilizó el análisis de supervivencia de Kaplan-Meier. Se aplicó el modelo de Cox para calcular el hazard ratio (HR) y el intervalo de confianza del 95% (IC95%) de supervivencia en función de las variables del estudio.

**Resultados:**

Se analizaron 116 personas mayores, la mayoría (n=83) tenían entre 60 y 69 años y fueron diagnosticados después de los 60 años (n=45). El riesgo de muerte fue mayor entre las personas mayores hospitalizadas durante el período estudiado (HR 4,83; IC95% 1,07; 21,79). Nueve personas murieron por causas relacionadas con el VIH y el tiempo medio de supervivencia fue de 76,5±48,5 meses. En el primer año de estudio, la probabilidad de supervivencia fue superior al 96%.

**Conclusión:**

La supervivencia varió desde menos de un mes hasta más de 195 meses. Entre los factores sociodemográficos y clínicos estudiados, sólo la ocurrencia de ingreso hospitalario mostró asociación significativa con la ocurrencia de muertes entre las personas mayores. Aunque el estudio se realizó en un solo servicio, estos resultados pueden contribuir para orientar estrategias de atención a las personas mayores con VIH.

## Introduction 

HIV infection and its progression to AIDS are a relevant public health problem. Infection affects different groups, with various forms of transmission: sexual, vertical and use of contaminated sharps (1-2).

HIV infections are increasing worldwide, whereby some important epidemics have been identified. Success or failure in combating infection depends on the path taken by current governments (3). In 2023, there were estimated to be 40 million people living with HIV globally (4). In Brazil, almost 37,000 HIV cases were reported in 2022, an increase of 17% when compared to the number reported in 2020 (5). Among the elderly, there was a 20% increase in the number of cases between 2015 and 2022. 

The increase in the number of cases of HIV infection in the elderly population may be associated with the lack of prevention campaigns aimed at safe sex practices among the elderly involving condom use, in addition to the existence of taboos about sexuality as age advances. Elderly people having insufficient knowledge about HIV infection may also be a factor (6).

Survival analyses of people living with HIV/AIDS in the context of the Brazilian regions and regarding the elderly are still incipient (7). This study aims to characterize, according to demographic and clinical aspects, the survival of elderly patients living with HIV in follow-up at a reference service in Jaboatão dos Guararapes, Pernambuco, Brazil.

## Methods 

### Design

This is a retrospective cohort study of HIV-infected elderly people in follow-up at a reference service in Jaboatão dos Guararapes, Pernambuco, Brazil. 

### Background

Jaboatão dos Guararapes is the second most populous municipality in the state of Pernambuco, with 644,037 inhabitants in 2022 (8), approximately 15% of whom were elderly. 

The study location, the Specialized Care Service, is a municipal outpatient care unit, a reference service for attending to and monitoring people living with HIV in the municipality. The service is comprised of a multidisciplinary team of professionals who provide care, guidance and advice to service users on prevention and treatment of sexually transmitted infections (9).

### Participants

The study included elderly people (≥60 years old) who began follow-up at the Specialized Service between January 2006 and December 2021 and who had follow-up at the service until December 2022. The study examined active cases and those closed due to transfer, treatment dropout or death during the period analyzed.

We excluded duplicate records or those with illegible information, cases whose medical records were missing and elderly patients undergoing follow-up care in private services.

### Data source and variables

Data were collected from the consultation follow-up and monitoring records held at the service, between October and December 2022. The primary outcome was death from causes related to HIV infection.

The following variables comprised the study: age (in years: 60-69, 70-79, >80), sex (female, male), age at diagnosis (in years: <60, >60), education (illiterate, elementary education, high school education and above), municipality of residence (Jaboatão dos Guararapes, other municipalities), living with a partner (yes, no), hospitalization (yes, no), contracted opportunistic infections (yes, no), HIV viral load (in copies/mL: <5,000, >5,000), treatment failure at last appointment (yes, no) and change of antiretroviral regimen (yes, no).

### Statistical methods

The service entry date for survival calculation was defined as the date monitoring started at the service. The final date was the date of death for patients who died and the date of the last consultation for patients whose final situation was active, transferred or treatment dropout. Survival time was defined as the duration of follow-up in months. 

The survival estimate for elderly people living with HIV/AIDS was calculated from a certain time T until the outcome, in this case, death from causes related to HIV infection. Censorsing was characterized by the final situation: transfer between services, deaths from other causes, treatment dropout and elderly people with active monitoring. 

The hazard ratio (HR) and 95% confidence interval (95%CI) of death were calculated based on the study variables using Cox regression. Survival was calculated using the Kaplan-Meier estimator and expressed as a survival curve, estimated according to the study variables. All variables with a p-value<0.2 were included in the multivariate analysis. The threshold for statistical significance was set at p-value<0.05. The analyses were performed using Stata 12.0.

## Results 

A total of 126 eligible records were identified, ten of which were excluded (9 records not located and 1 follow-up at a private service), so that 116 elderly people were included in the study. 

The majority (n=83) were 60 to 69 years old and male (n=60). Diagnosis occurred after the age of 60 (n=45), 73 had low education levels, 102 lived in Jaboatão dos Guararapes and 62/115 lived alone (Table 1). 

**Table 1:** Frequency, incidence ratio per 10,000 elderly people, hazard ratio (HR) and 95% confidence interval (95%CI) for death due to HIV according to study variables. Jaboatão dos Guararapes, Pernambuco, Brazil, 2006-2021 (n=116)

Variable	n (%)	Incidence ratio	HR (95%CI)	p-value
Age (years)				
60-69	83 (71.6)	9.26	1.00	
70-79	30 (25.9)	10.03	1.05 (0.21;5.23)	0.952
>80	3 (2.6)	31.21	3.97 (0.46;34.11)	0.208
Sex				
Female	56 (48.3)	7.32	1.00	
Mal	60 (51.7)	12.79	1.76 (0.44;7.05)	0.425
Age at time of diagnosis (years)				
<60	71 (61.2)	7.7		
>60	45 (38.8)	17.41	2.64 (0.64;10.91)	0.179
Schoolinga				
Illiterate	22 (20.8)	19.01	1.00	
Elementary education	51 (48.1)	5.00	0.28 (0.05;1.70)	0.169
High school education and above	33 (31.1)	11.4	0.56 (0.11;2.82)	0.485
Municipality of residence				
Jaboatão dos Guararapes	102 (87.9)	10.43	1.00	
Other municipalities	14 (12.1)	8.92	0.79 (0.98;6.35)	0.825
Lives with partner^b^				
Yes	53 (46.1)	9.63	1.00	
No	62 (53.9)	10.78	1.25 (0.33;4.70)	0.745
Hospitalization^b^				
Yes	19 (16.5)	24.11	3.15 (0.78;12.71)	0.107
No	96 (83.5)	7.95		
Opportunistic infections^c^				
Yes	33 (29.2)	7.52	1.00	
No	80 (70.8)	11.61	1.49 (0.31;7.23)	0.622
Last viral load (copies/mL)^d^				
<5,000	15 (13.8)	7.51	1.00	
>5,000	4 (3.7)	31.64	5.77 (0.35;94.65)	0.220
Undetectable	90 (82.6)	8.58	1.31 (0.16;10.92)	0.804
Failure at last consultation^a^				
Yes	22 (21.0)	14.05	1.00	
No	83 (79.0)	8.07	0.64 (0.15;2.74)	0.550
Change of regimen^b^				
Yes	63 (54.8)	6.66	1.00	
No	52 (45.2)	17.96	2.97 (0.75;11.81)	0.121

Notes: ^a^10 records excluded due to missing information for this variable; ^b^1 record excluded due to missing information for this variable; ^c^3 records excluded due to missing information for this variable; ^d^ 7 records excluded due to missing information for this variable.

The majority had not been hospitalized (n=96)and did not present opportunistic infections (n=80). Four elderly people had a viral load above 5,000 copies/mL at the last consultation, 83 had no treatment failure and 63 had changed their antiretroviral regimen during the follow-up period. In the univariate analysis, no factors associated with death due to HIV were identified (Table 1).

Hospitalization among the elderly increased the risk of death (HR 4.83; 95%CI 1.07; 21.79) (Table 2). Of the total number of elderly people in the study group, nine died from HIV-related causes. Average survival time of the elderly people monitored was 76.5±48.5 months, ranging from 0.63 months to 195.6 months.

**Table 2:** Hazard ratio (HR) and 95% confidence interval (95%CI) for death due to HIV according to study variables. Jaboatão dos Guararapes, Pernambuco, Brazil, 2006-2021 (n=116)

Variable	HR (95%CI)	p-value
Age at time of diagnosis (years)		
<60	1.00	
>60	2.70 (0.49; 14.94)	0.255
Schooling		
Illiterate	1.00	
Elementary education	0.35 (0.05; 2.32)	0.274
High school education and above	0.75 (0.12; 4.82)	0.760
**Hospitalization**		
Yes	4.83 (1.07; 21.79)	0.041
No	1.00	
Change of regimen		
Yes	1.00	
No	2.34 (0.43; 12.73)	0.326

**Figure 1:**
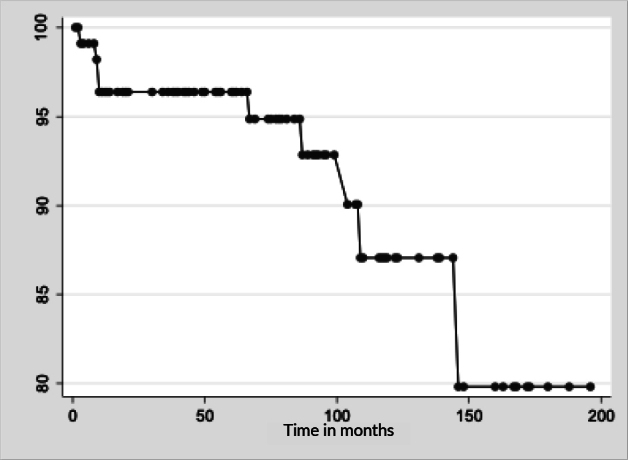
Probability of survival (%) of elderly people living with HIV over time. Jaboatão dos Guararapes, Pernambuco, Brazil, 2006-2021

In the first 12 months studied, there were four deaths with underlying causes related to HIV infection and the probability of survival was 96.4% (95%CI 90.68; 98.63) (Figure 1).

## Discussion 

During the period studied, the majority of elderly people monitored were between 60 and 69 years old, male, had been diagnosed with HIV infection under the age of 60, had no partners and had low education levels. The majority of the elderly people had not been hospitalized due to complications related to HIV infection or opportunistic infections, had a low viral load according to the last test recorded on their medical records, had no treatment failure and had to change their treatment regimen at least once. Average survival time was greater than 76 months. 

It can be seen that males have always been among those most vulnerable to HIV infection and although the phenomenon of feminization has occurred in the history of the epidemic, the proportion of men living with HIV is still greater than the proportion of women, as found in this study. In relation to age group, Brazil (10) has seen a sharp increase in cases of infection among the elderly.

Another important factor was that the majority of elderly people were between 60 and 69 years old. These can be classified according to age group into youngest-old people (11) and those who were diagnosed as having HIV infection under 60 years old. The greater proportion of elderly people diagnosed before the age of 60 demonstrates that reaching old age is the result of progress in access to antiretroviral therapy, which has been a right guaranteed by Brazil since 1996. The Ministry of Health (12) recognizes that increased survival, as a result of the use of antiretroviral drugs, has enabled many people living with HIV to reach old age.

However, almost 40% of the elderly people monitored were diagnosed over the age of 60, which may, on the one hand, be a reflection of the increase in the occurrence of infection in this age group, as identified by other authors (13-14), or difficulty in accessing services, which delays diagnosis and compromises care, as identified in a survey in the state of Alagoas (15). Furthermore, studies show that among the elderly requesting an HIV test only happens in the late stage of the disease, after the possibility of other diseases has been discarded (16-17).

 We also found that a greater proportion of elderly people are resident in the municipality of Jaboatão dos Guararapes, live alone, without partners and have low education levels. A study (18) about family and care of elderly people living with HIV revealed that positive HIV diagnosis among people aged 60 or over emphasizes the importance of care and a support network due to the need to rebuild bonds that break down following diagnosis. 

Furthermore, poor access to education can influence older people’s understanding of the risks of the disease and prevention methods (19). In addition, a low level of education is associated with poorer living conditions, food, housing, transportation, access to health services in general and social discrimination. Survival and adherence to antiretroviral treatment have been associated with lower levels of education (20).

In relation to clinical characteristics, we found a greater proportion of elderly people with undetectable viral loads, which may be a result of good adherence to treatment and few treatment failures. It is known that an undetectable viral load and a T-CD4 lymphocyte count above 350 favor (21) patients having a better defense response against infectious agents that can affect them, improving morbidity and mortality rates associated with the outcomes of opportunistic infections. 

Another aspect found in the results was that the majority of elderly people had not had opportunistic infections, although a small proportion of them were affected by these diseases. It is important to highlight that in addition to a high viral load and a decrease in CD4+ T cells, aspects related to socio-environmental conditions, can contribute to the emergence of opportunistic infections in immunocompromised individuals, such as water supply, sanitation, hygiene habits, having pets, among other contexts (22).

Regarding changes in treatment regimens, just over half changed antiretroviral regimens at least once during the period assessed. A survey (23) conducted with people living with HIV in the state of Ceará highlights that it is essential to take into account that any regimen change must be analyzed carefully, in order to maintain the safety and effectiveness of the treatment.

Occurrence of hospitalization was the only variable that remained in the regression model with significant association with the survival of the elderly. It was found that the largest proportion of elderly people did not have a record of hospitalization due to complications related to HIV infection, and those who had not been hospitalized during the study period had a higher survival rate. 

Therefore, hospitalization may be the result of treatment failure or non-adherence to treatment. This shows that the results corroborate the findings of a case-control study in a municipality in the state of São Paulo (24), which found that some programmatic vulnerabilities, such as irregular use of antiretrovirals, treatment dropout, not attending clinical follow-up appointments, are risk factors for hospitalization due to HIV/AIDS. 

Regarding the average survival time found in the study, despite the increase in HIV cases among the elderly, it can be seen that the universal supply of antiretroviral drugs contributed to greater survival (25). Age at diagnosis is an important predictor of survival. People diagnosed with HIV in younger age groups had a significantly higher average survival rate than those who were older. The age factor was found to be a negative indicator for those diagnosed when they were in the 41-60 and over 60 age groups, as they had lower mean and median survival rates (26).

It is essential to conduct studies on mortality due to HIV/AIDS. A survey of deaths from HIV/AIDS-defining and non-HIV/AIDS-defining illnesses in Brazil between 2000 and 2018 demonstrated that the existence of high mortality rates due to defining diseases may be a reflection of inequalities regarding death due to this health condition (27).

Among the limitations of this study, the use of data from medical records, which were not always well filled out and lacked information, hindered the analysis of some variables, such as income, education and adherence to treatment. However, this research provides support that can contribute to identifying the need to implement new sources of information monitoring for providing care to users of the Specialized Care Service. 

Future research, involving more health centers and other territories, may point to new findings related to the context of health policy and the planning of specific interventions to improve the quality of life of elderly people living with HIV.

This research revealed the survival characteristics of the elderly in follow-up that Service we studied. In the first year of study, probability of survival was greater than 96%. Survival ranged from less than one month to more than 195 months (more than 16 years). Among the sociodemographic and clinical factors studied, only occurrence of hospitalization showed significant association with the survival of the elderly. The elderly people monitored were mostly men, were in the youngest-old age group, were diagnosed with HIV before the age of 60, lived alone and had low education levels. Most of them had not been hospitalized, nor did they present opportunistic infections during the period analyzed. Although the study was conducted in just one Service, these results can contribute to the targeting of care strategies for elderly people living with HIV.
