# Supplementary material for: Survival of elderly people living with the human immunodeficiency virus in a municipality in Northeast Brazil: a retrospective cohort, 2006-2021
Source: Epidemiol Serv Saude. 2025 Mar 31;34:e20240071. doi: 10.1590/S2237-96222025v34e20240071.en (PMC11967166; doi:10.1590/S2237-96222025v34e20240071.en)
Supplement: Translation

# Translation: Sobrevida de pessoas idosas vivendo com o vírus da imunodeficiência humana em um município do nordeste brasileiro: coorte retrospectiva, 2006-2021

**DOI:** 10.1590/S2237-96222025v34e20240071.pt

**Authors:** Julianne Damiana da Silva Vicente, Cristine Vieira do Bonfim, Jessyka Mary Vasconcelos Barbosa, Vanessa de Lima Silva, Albanita Gomes da Costa de Ceballos, Gabriella Morais Duarte Miranda

**Affiliations:** 1 Universidade Federal de Pernambuco, Programa de Pós-Graduação em Saúde Coletiva, Recife, PE, Brasil; 2 Fundação Joaquim Nabuco, Diretoria de Pesquisas Sociais, Recife, PE, Brasil; 3 Ministério da Saúde, Brasília, DF, Brasil; 4 Universidade Federal de Pernambuco, Programa de Pós-graduação em Gerontologia, Recife, PE, Brasil; 5 Universidade Federal de Pernambuco, Programa de Pós-graduação em Saúde Coletiva, Recife, PE, Brasil

**Keywords:** Análise de Sobrevida, Infecções por HIV, Idoso, Perfil de Saúde, Estudos de Coortes

## Abstract

**Objetivo:**

Caracterizar a sobrevida de idosos com infecção por HIV, acompanhados no serviço de referência de Jaboatão dos Guararapes, Pernambuco.

**Métodos:**

Trata-se de uma coorte retrospectiva de idosos que iniciaram o acompanhamento entre 2006 e 2021. Empregou-se a análise de sobrevivência de Kaplan-Meier. Aplicou-se o modelo de Cox para calcular o hazard ratio (HR) e o intervalo de confiança de 95% (IC95%) da sobrevida pelas variáveis do estudo.

**Resultados:**

Foram analisados 116 idosos, a maioria (n=83) tinha 60-69 anos de idade e foram diagnosticados após os 60 anos (n=45). O risco de morte foi maior entre os idosos que apresentaram internação no período estudado (HR 4,83; IC95% 1,07; 21,79). Nove pessoas faleceram por causas relacionadas ao HIV e o tempo médio de sobrevida foi 76,5±48,5 meses. No primeiro ano de estudo, a probabilidade de sobrevivência foi superior a 96%.

**Conclusão:**

A sobrevida variou de menos de um mês a mais de 195 meses. Dentre os fatores sociodemográficos e clínicos estudados, somente a ocorrência de internação hospitalar apresentou significativa associação com a ocorrência de óbitos entre os idosos. Embora o estudo tenha sido realizado em um único serviço, esses resultados podem contribuir para o direcionamento das estratégias de cuidado aos idosos com HIV.

## Introdução 

A infecção por HIV e a evolução para aids constitui relevante problema para a saúde pública. A infecção acomete diversos grupos, com várias formas de transmissão: sexual, vertical e o uso de perfurocortantes contaminados (1-2).

As infecções pelo HIV estão aumentando no mundo, com a identificação de algumas importantes epidemias. O sucesso ou a falha no combate à infecção depende do caminho trilhado pelos governos atuais (3). Em 2023 estimaram-se 40 milhões de pessoas vivendo com o HIV no mundo (4). No Brasil foram notificados em 2022 quase 37 mil casos de HIV, com crescimento de 17% quando comparado ao número de notificações realizadas em 2020 (5). Entre os idosos, observou-se um aumento de 20% no número de casos entre 2015 e 2022. 

A ampliação do número de casos de infecção pelo HIV na população idosa pode estar associada à escassez de campanhas preventivas direcionadas à prática sexual segura entre idosos com a utilização de preservativos, além da existência de tabus sobre a sexualidade no envelhecimento. Conhecimento insuficiente de idosos sobre essa infecção também pode ser um fator (6).

Análises de sobrevida de pessoas vivendo com HIV/aids no contexto regional brasileiro e para os idosos, ainda são incipientes (7). Esse estudo tem por objetivo caracterizar, segundo aspectos demográficos e clínicos, a sobrevida de pacientes idosos com HIV acompanhados em um serviço de referência em Jaboatão dos Guararapes, Pernambuco.

## Métodos 

Delineamento

Trata-se de um estudo de coorte retrospectiva de idosos infectados pelo HIV acompanhados em um serviço de referência em Jaboatão dos Guararapes, Pernambuco. 

Contexto

Jaboatão dos Guararapes é o segundo município mais populoso de Pernambuco, com 644.037 habitantes em 2022 (8), dos quais aproximadamente 15% eram idosos. 

O local do estudo, Serviço de Atendimento Especializado é uma unidade assistencial de caráter ambulatorial municipal, referência no acolhimento e acompanhamento de pessoas com HIV no município. O serviço é composto por uma equipe multidisciplinar de profissionais que realizam o atendimento, orientação e aconselhamento aos usuários sobre prevenção e tratamento das infecções sexualmente transmissíveis (9).

Participantes

Foram incluídos os idosos (≥60 anos) que iniciaram o acompanhamento no Serviço Especializado entre janeiro de 2006 a dezembro de 2021 e que foram seguidos pelo serviço até dezembro de 2022. Consideraram-se os casos ativos e os encerrados por motivo de transferência, abandono de tratamento ou óbito no período de análise.

Registros duplicados ou com informações ilegíveis, prontuários não encontrados e pacientes idosos que realizam acompanhamento em rede particular foram excluídos.

### Fonte de dados e variáveis

Os dados foram coletados nos prontuários de acompanhamento e monitoramento de consulta do serviço, entre outubro e dezembro de 2022. O desfecho primário foi o óbito por causas relacionadas à infecção por HIV.

As seguintes variáveis compuseram o estudo: idade (em anos: 60-69, 70-79,>80), sexo (feminino, masculino), idade no momento do diagnóstico (em anos: <60, >60), escolaridade (analfabeto, ensino fundamental, ensino médio e mais), município de residência (Jaboatão dos Guararapes, outros municípios), residência com companheiro (sim, não), internações hospitalares (sim, não), infecções oportunistas adquiridas (sim, não), carga viral de HIV (em cópias/mL: <5.000, >5.000), falha na última consulta (sim, não) e troca de esquema do antirretroviral (sim, não).

### Métodos estatísticos

Foi definida como data de entrada para cálculo da sobrevida a data de início do acompanhamento no serviço. Como data final, a data do óbito para os pacientes que foram a óbito e a data da última consulta para os pacientes com situação final estabelecida como ativos, transferência ou abandono de tratamento. O tempo de sobrevida foi definido como a duração de seguimento em meses. 

A estimativa de sobrevida dos idosos com HIV/aids se deu a partir de um determinado tempo T até a ocorrência do desfecho, no caso, o óbito por causas relacionadas à infecção. A censura caracterizou-se pela situação final estabelecida pela transferência entre serviços, óbitos por outras causas abandono do tratamento e idosos com acompanhamento ativo. 

Calculou-se *hazard ratio* (HR) e intervalo de confiança de 95% (IC95%) do óbito pelas variáveis do estudo por meio de regressão de Cox. O cálculo da sobrevida foi realizado pelo estimador de Kaplan-Meier e expresso em curva de sobrevida, estimada de acordo com as variáveis do estudo. Todas as variáveis com p-valor<0,2 foram incluídas na análise multivariada. O limite para significância estatística foi estabelecido como p-valor<0,05. As análises foram realizadas no programa Stata 12.0.

## Resultados 

Foram identificados 126 prontuários elegíveis e 10 foram excluídos (9 prontuários não localizados e 1 acompanhamento na rede privada), totalizando 116 idosos incluídos. 

A maioria (n=83) tinham 60 a 69 anos de idade e eram do sexo masculino (n=60). O diagnóstico aconteceu após os 60 anos (n=45), 73 tinham baixa escolaridade, 102 residiam em Jaboatão dos Guararapes e 62/115 viviam sozinhos (Tabela 1). 

**Tabela 1:** Frequência, razão de incidência por 10 mil idosos, *hazard ratio* (HR) e intervalo de confiança de 95% (IC95%) do óbito por HIV pelas variáveis do estudo. Jaboatão dos Guararapes, 2006-2021 (n=116)

Variável	n (%)	Razão de incidência	HR (IC95%)	p-valor
Idade (anos)				
60-69	83 (71,6)	9,26	1,00	
70-79	30 (25,9)	10,03	1,05 (0,21;5,23)	0,952
>80	3 (2,6)	31,21	3,97 (0,46;34,11)	0,208
Sexo				
Feminino	56 (48,3)	7,32	1,00	
Masculino	60 (51,7)	12,79	1,76 (0,44;7,05)	0,425
Idade no momento do diagnóstico (anos)				
<60	71 (61,2)	7,7		
>60	45 (38,8)	17,41	2,64 (0,64;10,91)	0,179
Escolaridade^a^				
Analfabeto	22 (20,8)	19,01	1,00	
Ensino Fundamental	51 (48,1)	5,00	0,28 (0,05;1,70)	0,169
Ensino médio e mais	33 (31,1)	11,4	0,56 (0,11;2,82)	0,485
Município de residência				
Jaboatão dos Guararapes	102 (87,9)	10,43	1,00	
Outros municípios	14 (12,1)	8,92	0,79 (0,98;6,35)	0,825
Vive com companheiro(a)^b^				
Sim	53 (46,1)	9,63	1,00	
Não	62 (53,9)	10,78	1,25 (0,33;4,70)	0,745
Internação^b^				
Sim	19 (16,5)	24,11	3,15 (0,78;12,71)	0,107
Não	96 (83,5)	7,95		
Infecções oportunistas^c^				
Sim	33 (29,2)	7,52	1,00	
Não	80 (70,8)	11,61	1,49 (0,31;7,23)	0,622
Última carga viral (cópias/mL)^d^				
<5.000	15 (13,8)	7,51	1,00	
>5.000	4 (3,7)	31,64	5,77 (0,35;94,65)	0,220
Indetectável	90 (82,6)	8,58	1,31 (0,16;10,92)	0,804
Falha na última consulta^a^				
Sim	22 (21,0)	14,05	1,00	
Não	83 (79,0)	8,07	0,64 (0,15;2,74)	0,550
Troca de esquema^b^				
Sim	63 (54,8)	6,66	1,00	
Não	52 (45,2)	17,96	2,97 (0,75;11,81)	0,121

Notas: ^a^Excluídos 10 registros por falta de informação nesta variável; ^b^Excluído 1 registro sem informação por falta de informação nesta variável; ^c^Excluídos 3 registros por falta de informação nesta variável; ^d^ Excluídos 7 registros por falta de informação nesta variável.

A maioria não foi internada (n=96) e não apresentou doenças oportunistas (n=80). Quatro idosos possuíam na última consulta um registro de carga viral superior a 5.000 cópias/mL, 83 não apresentavam falha terapêutica e 63 tiveram troca do esquema do antirretroviral no período de seguimento. Na análise univariada não foram identificados fatores associados ao óbito por HIV (Tabela 1).

A internação entre os idosos aumentou o risco de óbito (HR 4,83; IC95% 1,07; 21,79) (Tabela 2). Do total de idosos do grupo de estudo, nove faleceram por causas relacionadas ao HIV. O tempo médio de sobrevida dos idosos acompanhados foi 76,5±48,^5^, variando de 0,63 meses a 195,6 meses.

**Tabela 2:** *Hazard ratio* (HR) e intervalo de confiança de 95% (IC95%) do óbito por HIV pelas variáveis do estudo. Jaboatão dos Guararapes, 2006-2021 (n=116)

Variável	HR (IC95%)	p-valor
Idade no momento do diagnóstico (anos)		
<60	1,00	
>60	2,70 (0,49; 14,94)	0,255
Escolaridade		
Analfabeto	1,00	
Ensino fundamental	0,35 (0,05; 2,32)	0,274
Ensino médio e mais	0,75 (0,12; 4,82)	0,760
Internação		
Sim	4,83 (1,07; 21,79)	0,041
Não	1,00	
Troca de esquema		
Sim	1,00	
Não	2,34 (0,43; 12,73)	0,326

Nos 12 primeiros meses estudados, ocorreram quatro óbitos com causas básica relacionadas à infecção por HIV e a probabilidade de sobrevivência foi 96,4% (IC 95% 90,68; 98,63) (Figura 1).

**Figura 1:** Probabilidade de sobrevida (%) de idosos com HIV pelo tempo. Jaboatão dos Guararapes, 2006-2021

## Discussão 

Durante o período estudado, a maioria dos idosos acompanhados tinha entre 60 e 69 anos, era do sexo masculino, havia recebido o diagnóstico da infecção com menos de 60 anos, não tinha companheiros e tinha baixa escolaridade. A maior proporção dos idosos não havia sido internada por complicações relacionadas à infecção, nem doenças oportunistas, possuía baixa carga viral no último exame registrado no prontuário, sem falha terapêutica e necessitou da troca, pelo menos uma vez, do esquema terapêutico. O tempo médio de sobrevida foi superior a 76 meses. 

Percebe-se que o sexo masculino sempre esteve entre aqueles com maior vulnerabilidade para a infecção e embora na história da epidemia tenha ocorrido o fenômeno da feminização, ainda há uma pequena proporção a mais de homens vivendo com HIV do que mulheres, como observado nesse estudo. Em relação à faixa etária, observa-se no país (10) o aumento dos casos de infecção entre os idosos de maneira vertiginosa.

Outro fator importante foi que a maioria dos idosos tinha entre 60 e 69 anos. Estes podem ser classificados conforme a faixa etária em idosos jovens (11) e tinham recebido o diagnóstico da infecção com menos de 60 anos. A maior proporção de pessoas idosas com diagnóstico realizado antes dos 60 anos demonstra que a chegada na terceira idade é fruto dos avanços ao acesso à terapia antirretroviral que é um direito assegurado pelo país desde 1996. O Ministério da Saúde (12) reconhece que a ampliação da sobrevida, como resultado do uso dos medicamentos antirretrovirais, possibilitou que muitas pessoas que vivem com HIV chegassem à terceira idade.

Entretanto, quase 40% dos idosos acompanhados tiveram o diagnóstico já na terceira idade, o que pode por um lado, ser reflexo do aumento que se observa na ocorrência da infecção nesta faixa etária, conforme identificado por outros autores (13-14) ou de uma dificuldade de acesso, que atrasa o diagnóstico e compromete o cuidado, como já identificado em uma pesquisa no estado de Alagoas (15). Para além disso, os estudos demonstram que a solicitação do exame para HIV entre idosos só acontece na fase tardia da doença, após a exclusão de outras doenças (16-17).

Observou-se também, que uma maior proporção de idosos são residentes do município do Jaboatão dos Guararapes, vivem sozinhos, sem companheiros e possuem baixa escolaridade. Estudo (18) sobre a família e o cuidado de idosos com HIV, revelou que o diagnóstico reagente para o HIV em pessoas com 60 anos ou mais, fortalece a importância do cuidado e uma rede de apoio para a necessidade de reconstrução de laços que se desfazem com o diagnóstico. 

Ademais, o pouco acesso à educação pode influenciar na compreensão dos riscos da doença e dos métodos de prevenção por parte das pessoas idosas (19). Além disso, a baixa escolaridade associa-se a piores condições de vida, alimentação, moradia, transporte, acesso a serviços de saúde no geral e discriminação social. A sobrevida e a adesão ao tratamento antirretroviral têm sido associadas a menores níveis de escolaridade (20).

Em relação às caraterísticas clínicas, observou-se a presença de uma maior proporção de idosos com cargas virais indetectáveis, que pode ser resultado da boa adesão ao tratamento e pouca falha terapêutica. Sabe-se que a carga viral indetectável e linfócitos T-CD4 acima de 350 são favoráveis (21) para que os pacientes apresentem uma melhor resposta da defesa contra os agentes infecciosos que os acometem, melhorando os índices de morbimortalidade associados aos desfechos das infecções oportunistas. 

Outro aspecto encontrado nos resultados, foi que a maioria das pessoas idosas não apresentaram infecções oportunistas, porém, uma pequena parcela foi acometida por essas doenças. É importante ressaltar que além de uma carga viral acentuada e diminuição das células TCD4+, aspectos relacionados às condições socioambientais podem contribuir para o surgimento de infecções oportunistas em indivíduos imunodeprimidos, como abastecimento de água, esgotamento sanitário, hábitos de higiene, manutenção de animal de estimação, entre outros contextos (22).

No que concerne à mudança de esquemas terapêuticos, pouco mais da metade apresentou troca de esquema do antirretroviral pelo menos uma vez durante o período avaliado. Uma pesquisa (23) realizada em pessoas com HIV no Ceará destaca que é imprescindível levar em consideração que qualquer troca deve ser bem analisada, a fim de manter a segurança e a eficácia do tratamento. 

A ocorrência de internação hospitalar foi a única variável que permaneceu no modelo de regressão com significativa associação com a sobrevida dos idosos. Observou-se que a maior proporção dos idosos não tinha o registro de internações por complicações relacionadas à infecção, e os que não apresentaram internações durante o tempo de estudo tiveram uma maior sobrevida. 

Logo, a internação pode ser o resultado de uma falha terapêutica ou da não adesão ao tratamento, isso mostra que os resultados corroboram com os achados de um estudo de caso controle em um município do estado de São Paulo (24), de que algumas vulnerabilidades programáticas, como a irregularidade no uso do antirretroviral, o abandono de tratamento, as ausências no acompanhamento clínico são fatores de risco para internação por HIV/aids. 

Sobre o tempo médio de sobrevida encontrado no estudo, apesar do aumento dos casos de HIV entre idosos, observa-se que a oferta universal de medicamentos antirretrovirais contribuiu para uma maior sobrevivência (25). A idade ao diagnóstico é um importante preditor de sobrevida. Pessoas diagnosticadas com HIV nas faixas etárias menores tiveram sobrevida média significativamente maior do que aquelas com mais idade. As faixas etárias de 41-60 anos e acima de 60 anos no diagnóstico apresentaram o fator idade como um indicador negativo, com média e medianas de sobrevida menores (26).

É fundamental realizar estudos sobre a mortalidade por HIV/aids. Uma pesquisa sobre os óbitos por doenças definidoras e não definidoras de HIV/aids no país entre 2000 e 2018, demonstrou que a existência de elevadas taxas de mortalidade por doenças definidoras pode ser reflexo das desigualdades na morte pelo agravo (27).

Entre as limitações deste estudo, a utilização de dados de prontuários, nem sempre bem preenchidos e com ausência de informações, prejudicou a análise de algumas variáveis, como renda, escolaridade e adesão ao tratamento. Todavia, esta pesquisa fornece subsídios que podem contribuir para identificar a necessidade de implantação de novas fontes de monitoramento de informações para o atendimento dos usuários do Serviço de Atenção Especializada. 

Futuras pesquisas, envolvendo mais unidades de saúde e outros territórios podem apontar novos achados relacionados ao contexto da política de saúde e ao planejamento de intervenções específicas para melhorar a qualidade de vida dos idosos com HIV.

A pesquisa evidenciou as características da sobrevida dos idosos acompanhados pelo Serviço estudado. No primeiro ano de estudo, a probabilidade de sobrevivência foi superior a 96%. A sobrevida variou de menos de um mês a mais de 195 meses (mais de 16 anos). Dentre os fatores sociodemográficos e clínicos estudados, somente a ocorrência de internação hospitalar apresentou significativa associação com a sobrevida dos idosos. Os idosos acompanhados eram em sua maioria homens, não longevos, que tiveram seus diagnósticos de HIV antes dos 60 anos, que moravam sozinhos e tinham baixa escolaridade. A maior parte deles não se internou, nem apresentou doenças oportunistas no período analisado. Embora o estudo tenha sido realizado em um único Serviço, esses resultados podem contribuir o direcionamento das estratégias de cuidado aos idosos com HIV.
